# Effects of Chronic Thermal Stress on the Physiology, Metabolism, Histology, and Gut Microbiota of Juvenile *Schizothorax grahami*

**DOI:** 10.3390/ani15182749

**Published:** 2025-09-20

**Authors:** Shuangqian Bai, Tingyin Li, Lingfu Kong, Baoliang Bi, Qing Hu

**Affiliations:** 1 Faculty of Animal Science and Technology, Yunnan Agricultural University, Kunming 650201, China; 2 Key Laboratory for Plateau Fishery Resources Conservation and Sustainable Utilization of Yunnan Province, Yunnan Agricultural University, Kunming 650201, China; 3 International College, Yunnan Agricultural University, Kunming 650201, China

**Keywords:** *Schizothorax grahami*, thermal stress, histological, metabolism, gut microbiota

## Abstract

Temperature is a critical factor influencing fish health and aquaculture success. This study investigates the physiological, histological, and microbiota responses of juvenile *Schizothorax grahami* to chronic thermal stress across six temperature treatments. We found that exposure to temperatures exceeding 27 °C resulted in complete mortality in juvenile *S. grahami*, with the upper thermal tolerance range between 24 °C and 27 °C. Chronic thermal stress caused a significant decline in serum glucose (Glu), triglycerides (TG), and total cholesterol (TC) at 24 °C, indicating rapid energy depletion, while reduced serum enzyme activity of catalase (CAT) and total superoxide dismutase (T-SOD) at 24 °C group further reflected a decrease in antioxidant capacity. Histological examination revealed adaptive intestinal villus hypertrophy with increased length and muscularis thickness at temperature under 24 °C. Furthermore, the relative abundance of *Cetobacterium* and Fusobacteriota suggested either adaptive responses or stress-related dysbiosis that may contribute to weakened host immune function. Overall, our findings highlight the vulnerability of juvenile *S. grahami* to rising temperatures, with moderate thermal stress inducing adaptive responses, while higher temperatures impair metabolism, gut integrity, microbial health, and may even lead to lethality. These results underscore the need for effective conservation strategies to protect this critically endangered species in the face of climate change.

## Simple Summary

*Schizothorax grahami* (Regan, 1904) is a representative species of Schizothoracine fish and has been classified as critically endangered (CR) by the International Union for Conservation of Nature (IUCN). This cold-water species is native to Southwest China, with its distribution spanning the Yunnan–Guizhou Plateau and the Tibetan Plateau—both ecologically sensitive regions highly vulnerable to human activities and climate change. Over the past decade, Southwest China has experienced frequent rainfall deficits and extreme high temperatures, severely impacting local populations and biodiversity, particularly native cold-water fish species. To evaluate the effects of climate-related thermal stress, we conducted a chronic thermal stress experiment on juvenile *S. grahami*. The results indicated an upper thermal tolerance range between 24 °C and 27 °C for this species. Moderate thermal stress elicited adaptive physiological responses, whereas higher temperatures significantly reduced energy reserves and impaired immune capacity in juveniles. This study provides the first comprehensive assessment of the effects of chronic thermal stress on juvenile *S. grahami*. Our findings offer an important scientific basis for developing targeted conservation measures, including habitat management and adaptive strategies, to protect this critically endangered species under ongoing climate change.

## 1. Introduction

Anthropogenic climate change, primarily driven by global warming, is causing a continuous rise in water temperatures across aquatic ecosystems, leading to widespread thermal stress on freshwater fish species [1,2]. Thermal stress can alter fish distribution patterns and community dynamics, with stenothermal species facing risks such as habitat loss and reduced reproductive success due to rising water temperatures and extreme temperature events [3]. For instance, the threatened longfin smelt (*Spirinchus thaleichthys* (Ayres, 1860)) exhibits limited tolerance to temperatures around 20 °C, indicating minimal capacity for adaptation to future warming. Studies in California have shown that this species experiences increased stress responses and mortality under elevated water temperatures, compounded by severe droughts in recent years [4]. A global assessment revealed that one-quarter of freshwater species, including fish, are at risk of extinction due to stressors such as temperature increases, habitat modification, and water extraction [5]. This issue is particularly severe in areas highly vulnerable to global warming, such as the Tibetan Plateau [6,7].

Temperature modulates enzyme activity in ectothermic fish, influencing nearly all physiological processes [8]. Thermal stress accelerates biochemical reactions, which can potentially impair the capacity of fish to maintain energy homeostasis over extended periods, consequently reducing growth rates and overall productivity [9]. Transcriptomic reprogramming in fish exposed to thermal stress entailed alternation in the expression of genes associated with heat shock response, apoptosis, intracellular signaling cascades, reactive oxygen species accumulation, inflammatory signaling, and various metabolic pathways [10]. While heat shock proteins (HSPs) and antioxidant enzymes help mitigate cellular damage, prolonged exposure can overwhelm these mechanisms, finally reducing immune function and increasing vulnerability to pathogen infections [11,12]. Thermal stress has been shown to alter the gut microbiota composition in species such as juvenile milkfish (*Chanos chanos* (Fabricius, 1775)), potentially affecting energy absorption and immune functions via intestines, and thereby influencing their long-term adaptation to elevated water temperatures [13,14]. Meanwhile, fish species exhibit diverse capacities for thermal tolerance and adaptation, with cold-water species often showing limited heat tolerance compared to their warm-water counterparts [15,16]. These adaptations are shaped by both genetic factors and environmental pressures, with regional differences influencing how species cope with temperature variations, especially under the threat of climate change.

The Tibetan Plateau, characterized by cold mountain streams and high altitudes, is particularly vulnerable to climate change impacts. Schizothoracine fish, like *Schizothorax grahami*, have evolved unique adaptations to survive in these extreme conditions, but are now threatened by rising temperatures and habitat degradation [6]. *S. grahami* is a representative species of Schizothoracine fish, which has been classified as critically endangered (CR) by International Union for Conservation of Nature (IUCN) due to habitat degradation and climate change impacts [17]. Although temperature tolerance studies on the congeneric species *Schizothorax prenanti* (Tchang, 1930) [18] and *Schizothorax kozlovi* (Nikolskii, 1903) [19] have been conducted, both revealing a high risk of mortality under thermal stress, no similar studies have been carried out for *S. grahami*, which inhabits an environment markedly different from those species. In the past decade, the primary habitat of *S. grahami* in Southwest China has experienced frequent super droughts, which have led to significant economic losses and ecological degradation, further endangering the survival of temperature-sensitive fish species in the region [20,21]. To assess the impacts of chronic thermal stress on juvenile *S. grahami*, we investigated its survival rate, metabolic activity, histological changes, and gut microbiota composition across a range of temperature conditions for 30 days. Understanding the physiological and microbial responses to chronic thermal stress provides a robust basis for evaluating the severity of heat-related threats to juvenile *S. grahami* in its native habitat. These findings will inform future research and contribute to the development of effective conservation strategies for *S. grahami* under the ongoing pressures of a warming climate.

## 2. Material and Methods

### 2.1. Fish and Experimental Design

Juvenile *S. grahami* were purchased from Kunming Tanghao Aquaculture Co., Ltd. (Kunming, China), and transported to the Aquaculture Laboratory at Yunnan Agricultural University. Prior to the formal experiment, the fish were temporarily feeding indoors with circulating water maintained at 15 °C for 7 days. A total of 360 fish, with an average initial body weight of 20.76 ± 3.08 g, were randomly divided into six groups, 15 °C (control group), 18 °C, 21 °C, 24 °C, 27 °C, and 30 °C, with three replicates per group and 20 fish per replicate in a 150 L tank with a recirculating water system. The temperature in the experimental tanks was raised to the preset level at a rate of 0.5 °C per hour, maintaining a temperature fluctuation within ±0.5 °C using heaters. The dissolved oxygen concentration was maintained at 6 mg/L across all temperature treatments by precise aeration devices. The feeding rate was set at 2% of the fish’s body weight. The photoperiod was set as 12 h light:12 h dark. Throughout the experiment, 20% of the water was replaced every two days with aerated water. The water quality maintained as follows: pH 7.8~8.2, ammonia nitrogen ≤ 0.2 mg/L, and nitrite ≤ 0.2 mg/L. The domestication experiment lasted for 30 days. All procedures conducted in this study followed the ethical guidelines for the Use of Animals in Research provided by Yunnan Agricultural University.

### 2.2. Sample Preparation

Due to their intolerance to 27 °C and 30 °C, all fish in these two temperature groups died on day 2 and day 13 of experiment, respectively. Therefore, samples were collected from the remaining fish in other four groups (control, 18 °C, 21 °C, and 24 °C) on day 30. From each replicate tank, three individual fish were randomly selected, yielding a total of nine biological replicates per group (n = 9). These samples were used for subsequent biochemical indicator measurement, RT-qPCR analysis, and Western blot analyses. For the microbiota analysis, three mid-intestinal samples from a single replicate tank were pooled to form one composite sample, and this procedure was repeated across all three replicate tanks, resulting in three pooled samples per group (n = 3 composite samples).

For serum extraction, 3 fish from each replicate tank were randomly anesthetized using 120 mg/L MS-222, and then blood was collected from the tail vein using a sterile syringe and transferred into 2 mL centrifuge tubes. After standing for 30 min at 4 °C, the serum was separated by centrifugation at 4 °C for 15 min and then stored at −80 °C for further biochemical analysis. The middle intestine was fixed in 4% paraformaldehyde solution for histological analysis. The liver and the remaining part of the middle intestine sample were frozen in liquid nitrogen and stored at −80 °C for the subsequent Western blot analysis, RT-qPCR assay, and microbiota analysis.

### 2.3. Serum Biochemical Indicator Measurement

Serum biochemical parameters, including triglycerides (TG), total cholesterol (TC), glucose (Glu), total antioxidant capacity (T-AOC), malondialdehyde (MDA), catalase (CAT), and total superoxide dismutase (T-SOD), were measured using commercial kits from the Nanjing Jiancheng Bioengineering Institute (Table S1), following the manufacturer’s instructions. Each parameter measurement was performed in triplicate from three independent tanks (n = 9).

### 2.4. Intestine Histological Analysis and Measurements

Middle intestine samples were fixed in 4% paraformaldehyde at room temperature for 5 days, and then dehydrated in ethanol, immersed in xylene, and embedded in paraffin. Sections (6 μm thick) were stained with hematoxylin and eosin and observed under a light microscope. Nine slides were prepared per group, with one representative view per slide selected for measurement. The slides were observed using a CX20 microscope (Sunny Optical Technology, Yuyao, China), and intestinal villi length, villi width, and intestinal muscularis thickness were measured and captured using the affiliated capture system. The representative images from each group were selected to demonstrate the effects to the intestinal structure caused by thermal stress. The count number of each villi parameter is shown in Table S2.

### 2.5. Western Blot Analysis

Total protein was extracted from the liver using RIPA lysis buffer (Sodium Deoxycholate, 1%; Triton X-100, 1%; SDS, 0.1%; NaCl, 150 nM; Tris, pH 7.4, 10 mM; PMSF, 1 mM) and separated by SDS-PAGE at 150 V for 10 min, followed by 180 V for 35 min. Proteins were transferred onto a PVDF membrane at 300 mA for 30 min. The membrane was then blocked for 2 h at room temperature and incubated with primary antibody overnight at 4 °C. After incubation with the HRP-conjugated secondary antibody for 1.5 h at room temperature, protein detection was performed using the BeyoECL Star kit (Beyotime Biotech, Shanghai, China). The details of primary and secondary antibodies used in this study are shown in Table S3. Western blot was performed in triplicate from three independent tanks (n = 9), and the original bands for each protein are shown in Figure S1. Images were captured using the ChemiDoc Imaging System (Bio-Rad, Hercules, CA, USA), and band intensities were quantified with ImageJ software (v.1.54). The relative expression of target proteins was normalized to GAPDH, and the intensity ratios of each band are presented in Table S4.

### 2.6. RT-qPCR Analysis

Total RNA was extracted from the liver of each sample using TRIeasy™ LS Total RNA Extraction Reagent (Yeasen Biotech, Shanghai, China). RNA integrity was verified by 1.5% agarose gel electrophoresis, while RNA purity and concentration were determined by NanoDrop (Thermo Fisher Scientific Inc., Waltham, MA, USA). Equal amounts of high-quality RNA from each sample were reverse-transcribed into cDNA using the Hifair^®^ III 1st Strand cDNA Synthesis SuperMix for qPCR (gDNA digester plus) kit (Yeasen Biotech, Shanghai, China). RT-qPCR was performed with the Hieff UNICON^®^ Universal Blue qPCR SYBR Green Master Mix kit (Yeasen Biotech, Shanghai, China) on a fluorescence qPCR instrument (Bio-Rad, Hercules, CA, USA) for quantitative gene expression analysis. Each 20 µL reaction contained 10 µL of 2× Universal Blue qPCR SYBR Green Master Mix, 0.4 µL of each forward and reverse primer (10 µM), 2 µL of cDNA template, and 7.2 µL of nuclease-free water. The thermal cycling conditions were as follows: initial denaturation at 95 °C for 2 min, followed by 40 cycles of 95 °C for 10 s and 60 °C for 30 s. Validated primer information for the RT-qPCR reactions is provided in Table S5. RT-qPCR was performed in triplicate from three independent tanks (n = 9). Gene expression was normalized to β-*actin*, and relative expression levels were calculated using the 2^−∆∆Ct^ method [22].

### 2.7. Gut Microbiota Analysis

Intestinal bacterial genomic DNA was extracted from middle intestine using the TIANamp Bacteria DNA Kit (TIANGEN Biotech, Beijing, China) according to the manufacturer’s instructions. The V4 region of the 16S rRNA gene was amplified from the extracted gDNA and sequenced using the Illumina NovaSeq 6000 system (Illumina, Inc., San Diego, CA, USA). For each group, three mid-intestinal samples from a single replicate tank were pooled to form one composite sample for 16S rRNA sequencing. This procedure was performed across all three replicate tanks, resulting in three composite samples per group (n = 3). Quality filtering, length trimming, and homopolymer truncation were carried out using Mothur (v. 1.44.0). The alpha diversity of ACE index was calculated with the Qiime2 (2020.6) package. Principal coordinate analysis (PCoA) was employed to evaluate beta diversity. Metagenomic analysis was further applied to evaluate the taxa with significant differences between the control group and the experimental groups.

### 2.8. Statistical Analysis

All data were expressed as mean ± SEM. The normality of the data distribution was assessed using the Kolmogorov–Smirnov test, and the homogeneity of variance was assessed using Levene’s test. All datasets met the assumptions required for subsequent statistical analyses. One-way analysis of variance (ANOVA) was performed for group comparisons, followed by Tukey’s post hoc test for multiple comparisons. Statistical analyses were conducted using SPSS 26 (IBM, Armonk, NY, USA), and a *p*-value of <0.05 was considered statistically significant.

## 3. Results

### 3.1. Effects of Chronic Thermal Stress on Survival of Juvenile S. grahami

**Figure 1:**
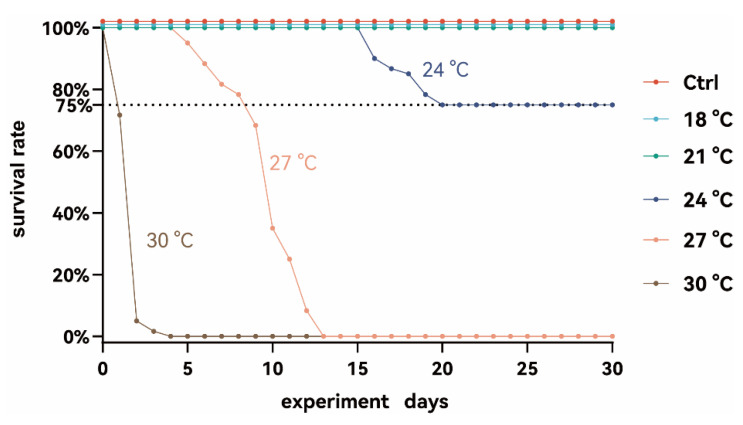
The survival rate of *S. grahami* in different temperature treatment groups.

A significant difference in survival rates was observed across the temperature treatments (Figure 1). All fish in the 30 °C and 27 °C groups died within 3 and 13 days, respectively. In the 24 °C group, mortality began on day 15 and continued until day 20, after which the survival rate stabilized at 75%. No further deaths occurred in this group after day 20. In contrast, no mortality was observed in the 15 °C, 18 °C, or 21 °C groups throughout the experiment. Consequently, subsequent measurements were conducted only on fish in the 15 °C, 18 °C, 21 °C, and 24 °C groups. Additionally, no significant differences in growth rates were observed among these groups across the whole experiment.

### 3.2. Effects of Chronic Thermal Stress on Serum Basic Biochemical Parameters and Antioxidant Enzyme Activities in Juvenile S. grahami

**Figure 2:**
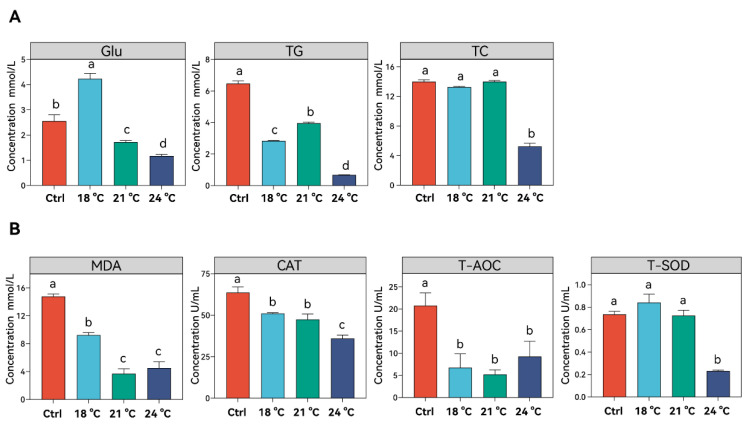
The serum basic biochemical parameters and antioxidant enzyme activities of *S. grahami* in different temperature treatment groups. (**A**) The concentration of glucose (Glu), triglyceride (TG), and total cholesterol (TC). (**B**) The concentration of malondialdehyde (MDA) and the activity of catalase (CAT), total antioxidant capacity (T-AOC), and total superoxide dismutase (T-SOD). Bars with different letters indicate significant differences (*p* < 0.05).

Thermal stress induced significant changes in serum biochemical parameters and antioxidant enzyme activities (Figure 2). Glu levels significantly increased at 18 °C but decreased markedly as temperatures rose from 21 °C to 24 °C (*p* < 0.05), even dropping below control levels (*p* < 0.05). TG levels significantly decreased in all treatment groups compared to the control, although a significant increase was observed when the temperature rose from 18 °C to 21 °C (*p* < 0.05), followed by a dramatic drop when the temperature further increased to 24 °C (*p* < 0.001). TC levels remained stable at 18 °C and 21 °C (*p* > 0.05) but significantly decreased at 24 °C (*p* < 0.05).

The main antioxidant system was also influenced by thermal stress (Figure 2B). MDA levels decreased in a temperature-dependent manner from 15 °C to 21 °C and remained stable with no significant difference when the temperature further increased to 24 °C (*p* > 0.05). CAT activity significantly decreased at 18 °C (*p* < 0.05), remained stable at 21 °C, and then significantly declined at 24 °C compared to the control (*p* < 0.05). T-AOC activity significantly decreased in all treatment groups compared to the control group (*p* < 0.05), with no significant differences observed among the treatment groups. T-SOD activity remained stable as the treatment temperature increased from 15 °C to 21 °C (*p* > 0.05), but it significantly decreased in the 24 °C group compared to the control (*p* < 0.05).

### 3.3. Effects of Chronic Thermal Stress on Middle Intestinal Structure in Juvenile S. grahami

**Figure 3:**
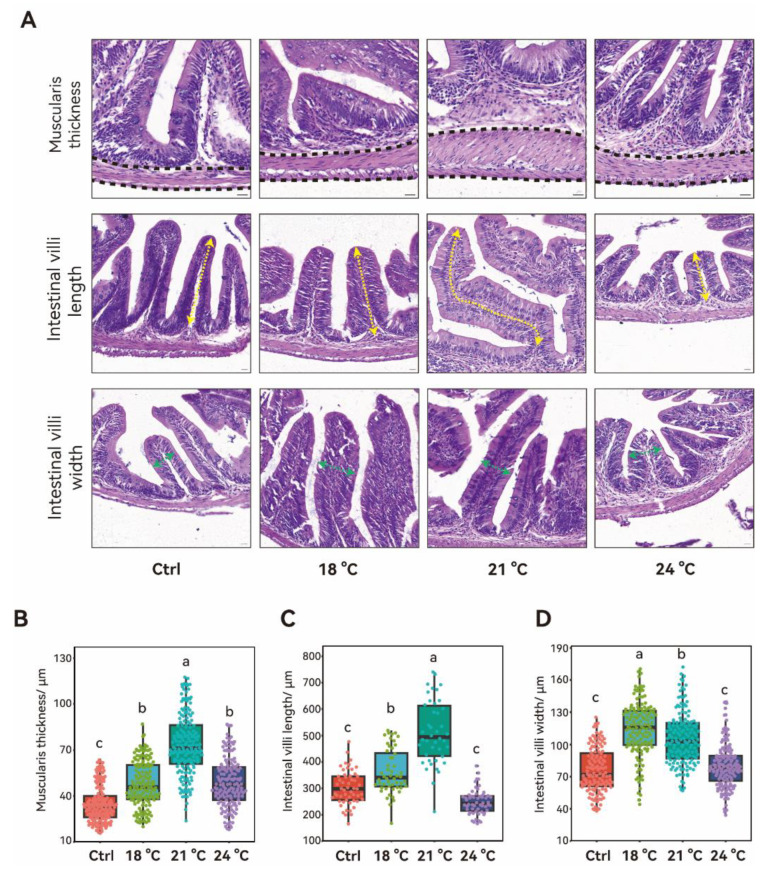
The middle intestinal structure of *S. grahami* in different temperature treatment groups. (**A**) Representative images of middle intestinal tissue showing muscularis thickness and intestinal villi length and width. Black dashed lines outline the muscularis thickness. Yellow and green arrows indicate villi length and width, respectively. Scale bar = 100 μm. (**B**) Quantification of muscularis thickness in *S. grahami*. (**C**) Quantification of middle intestinal villi length in *S. grahami*. (**D**) Quantification of middle intestinal villi width in *S. grahami*. Bars with different letters indicate significant differences (*p* < 0.05).

Thermal stress significantly affected the structure of the middle intestine, particularly in the muscularis thickness and intestinal villi length and width (Figure 3A). The thickness of the intestinal muscularis increased progressively from 15 °C to 21 °C in a temperature-dependent manner (*p* < 0.05), followed by a significant decline at 24 °C (Figure 3B). Despite this decrease, the thickness at 24 °C remained significantly greater than that of the control group (*p* < 0.05). Villi length significantly increased from 15 °C to 21 °C (*p* < 0.05) and returned to control levels at 24 °C (*p* > 0.05) (Figure 3C). Villus width showed a distinct pattern, peaking at 18 °C (*p* < 0.05) and then decreasing significantly as the temperature rose from 18 °C to 24 °C (*p* < 0.05). At 24 °C, villus width was not significantly different from the control group (*p* > 0.05).

### 3.4. Effects of Chronic Thermal Stress on Hepatic Stress- and Inflammation-Related Protein Expression in Juvenile S. grahami

**Figure 4:**
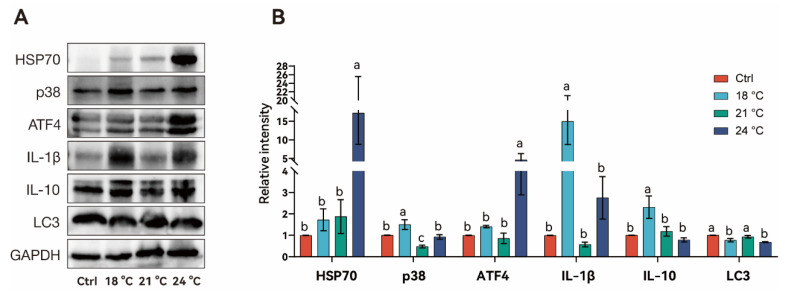
The related hepatic protein expression level of *S. grahami* in different temperature treatment groups. (**A**) Western blot analysis showing protein expression levels of HSP70, p38, ATF4, IL-1β, IL-10, and LC3. GAPDH is used as a loading control. (**B**) Quantitative of relative protein expression intensities normalized to GAPDH. Bars with different letters indicate significant differences (*p* < 0.05).

Stress- and inflammation- related proteins were significantly influenced under thermal stress (Figure 4). Heat shock protein 70 (HSP 70) levels significantly increased around 17-fold at 24 °C compared to the control group (*p* < 0.001). p38 expression was significantly higher at 18 °C (*p* < 0.05), but returned to control levels at 24 °C (*p* > 0.05). Activating transcription factor 4 (ATF4) expression followed a similar pattern to HSP70 with extreme increases at 24 °C. Interleukin-1 beta (IL-1β) expression significantly increased at 18 °C (*p* < 0.05) but returned to control levels at 21 °C. Although IL-1β level increased at 24 °C, no significant statistical difference was observed compared to the control (*p* > 0.05). Interleukin-10 (IL-10) expression significantly increased at 18 °C, but then significantly decreased in the 21 °C and 24 °C group, showing no difference compared to the control (*p* > 0.05). Microtubule-associated protein 1 light chain 3 (LC3) expression fluctuated under thermal stress, with a significant decrease in the 18 °C group (*p* < 0.05), followed by a significant increase in the 21 °C group, though it remained lower than in the control group.

### 3.5. Effects of Chronic Thermal Stress on Hepatic Gene Expression in Juvenile S. grahami

**Figure 5:**
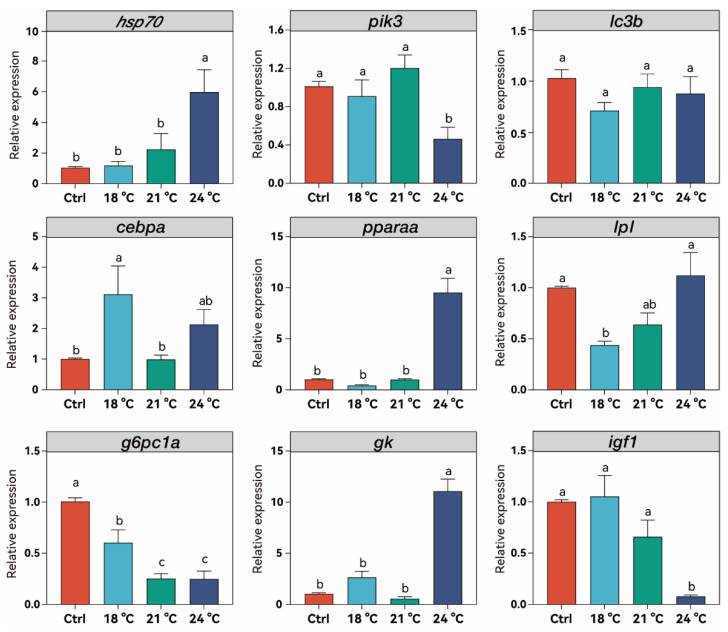
The related hepatic gene expression level of *hsp70*, *pik3*, *lc3b*, *cebpa*, *pparaa*, *lpl*, *g6pc1a*, *gk*, and *igf1* in *S. grahami* in different temperature treatment groups. Bars with different letters indicate significant differences (*p* < 0.05).

Hepatic genes related to stress response and cell growth were significantly changed under thermal stress. The expression pattern of *hsp70* was consistent with its protein expression, with a 6.04-fold increase at 24 °C compared to the control (*p* < 0.001). Gene expression of phosphoinositide-3-kinase (*pik3*) was not influenced at the lower temperature but significantly decreased at 24 °C compared to the control (*p* < 0.05). Microtubule-associated protein 1 light chain 3 beta (*lc3b*) gene expression showed no significant difference across all treatment groups compared to the control (*p* < 0.05). Hepatic genes related to lipid and glucose metabolism were significantly affected by thermal stress (Figure 5). CCAAT/enhancer binding protein a (*cebpa*) expression significantly increased at 18 °C (*p* < 0.05) and then returned to control levels at 21 °C. Although expression increased again at 24 °C, no significant difference was observed compared to either the control or the 21 °C group. Peroxisome proliferator-activated receptor alpha (*pparaa*) expression remained unchanged at 18 °C and 21 °C, but significantly increased at 24 °C, nearly 10-fold compared to the control (*p* < 0.001). Lipoprotein lipase (*lpl*) expression significantly decreased at 18 °C (*p* < 0.05), followed by a gradual increase at 21 °C and 24 °C, returning to the control levels (*p* > 0.05). Gene expression of glucose-6-phosphatase (*g6pc1a*) significantly decreased in a temperature-dependent manner from 15 °C to 21 °C (*p* < 0.05), without further change at 24 °C compared to the 21 °C group (*p* > 0.05). Gene expression of glucokinase (*gk*) remained unchanged at 18 °C and 21 °C, but it significant increased 11.07-fold compared to the control group (*p* < 0.001). Insulin like growth factor 1 (*igf1*) expression showed no significant change at the lower temperature in the 18 °C and 21 °C groups, but it significantly decreased at 24 °C compared to the control (*p* < 0.001).

### 3.6. Effects of Chronic Thermal Stress on Gut Microbiota in Juvenile S. grahami

**Figure 6:**
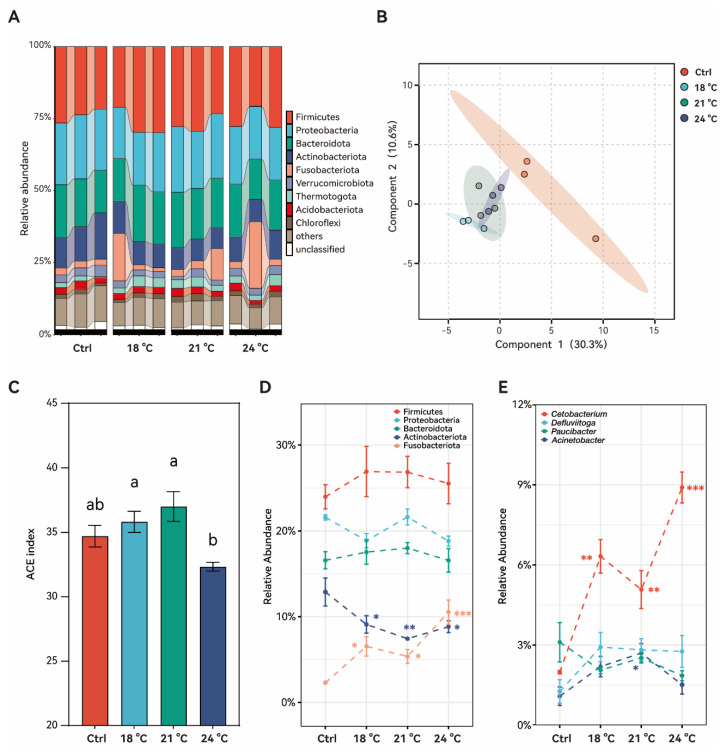
Composition of the gut microbiota of *S. grahami* in different temperature treatment groups. (**A**) Relative abundances of gut microbiota at the phylum level across different treatment groups. (**B**) Principal coordinate analysis (PCoA) based on Bray–Curtis distances at the phylum level. (**C**) ACE index representing α-diversity across different groups. Bars with different letters indicate significant differences (*p* < 0.05). (**D**) Relative abundance of the top five phyla across different groups. Asterisks indicate significant differences compared to the control (* *p* < 0.05, ** *p* < 0.01, *** *p* < 0.001). (**E**) Relative abundance of the top four genera across different groups. Asterisks indicate significant difference compared to the control (* *p* < 0.05, ** *p* < 0.01, *** *p* < 0.001).

Thermal stress altered the composition of the gut microbiota (Figure 6). Relative abundance at the phylum level is showed in Figure 6A. On the basis of Bray–Curtis distances, the PCoA was conducted to assess differences in phylum composition among groups (Figure 6B). Notably, the thermally stressed groups clustered together and were clearly separated from the control group, indicating distinct microbial community structures under thermal stress. Alpha diversity of the ACE index remained stable in the 18 °C and 21 °C groups (*p* > 0.05). However, a significant decrease was detected in the 24 °C group compared to the 18 °C and 21 °C groups (*p* < 0.05), although it still did not differ significantly from the control group (*p* > 0.05) (Figure 6C).

At the phylum level, the relative abundances of the dominant Firmicutes, Proteobacteria, and Bacteroidota were not significantly affected by increasing temperature (*p* > 0.05). In contrast, the relative abundance of Actinobacteriota significantly decreased under thermal stress, while Fusobacteriota showed a significant increase compared to the control group, especially in the highest temperature group, at 24 °C (*p* < 0.001) (Figure 6D). At the genus level, the dominant *Cetobacterium* showed a significant increase under thermal stress, even reaching almost three-fold at 24 °C compared to the control group (*p* < 0.001). In contrast, *Acinetobacter* significantly increased only at 21 °C (*p* < 0.05), followed by a decrease at 24 °C to a level comparable to the control (*p* > 0.05) (Figure 6E).

## 4. Discussion

### 4.1. Exposure to Temperatures Above 30 °C Induces Acute Lethality in Juvenile S. grahami

*S. grahami* is a cold-water fish species with a narrow thermal tolerance range. In the present study, the critical thermal maximum for juvenile *S. grahami* was determined to be approximately 27 °C, with a significant physiological response observed in the surviving individuals at 24 °C, thereby establishing its upper thermal tolerance range between 24 °C and 27 °C. Exposure to temperatures above 27 °C resulted in complete mortality, with acute mass death occurring at 30 °C. Similarly, a previous study on juvenile *S. kozlovi* identified the upper optimal growth temperature as 20.6 °C, with mortality increasing sharply at temperatures exceeding 32.8 °C [19]. In contrast, the thermal tolerance for juvenile *S. prenanti* was approximately 22 °C [18]. This comparison highlights the high thermal sensitivity shared among *Schizothorax* species, particularly during early developmental stages.

### 4.2. Chronic Exposure to Thermal Stress Leads to Rapid Energy Depletion and Disruption of the Antioxidant System in Juvenile S. grahami

Elevated temperatures accelerate physiological processes in fish, which may contribute to the increase in basal energy demand and greater energy expenditure on maintenance [23]. In addition to the significant activation of stress response genes and proteins, such as HSP70 and ATF4, there is a notable impact on energy metabolism. In this study, serum concentration of both Glu and TG significantly decreased in the 24 °C group, indicating increased energy consumption under thermal stress. And the hepatic genes related to glucose and lipid metabolism, such as *g6pc1a*, *gk*, *cebpa*, and *pparaa*, showed expression patterns consistent with these serum changes. A previous study on largemouth bass (*Micropterus salmoides* (Lacepède, 1802)) showed increased glycolysis and reduced gluconeogenesis under heat stress, leading to rapid energy depletion and reduced glycogen storage [24]. This metabolic reprogramming supports immediate energy demand but may reduce long-term reserves. Similar results were also found in common carp (*Cyprinus carpio* (Linnaeus, 1758)), in which triglycerides in the liver and heart decrease under thermal stress due to suppressed lipase activity, while related lipoprotein gene expression was also influenced to support altered lipid processing [25]. The significant increase in hepatic *gk*, *igf1*, and *pparaa* in juvenile *S. grahami* was accompanied by significant reductions in serum triglycerides and cholesterol [26,27]. These findings suggest that serum glucose, triglycerides, and cholesterol respond differentially to varying intensities of thermal stress in juvenile *S. grahami*.

Thermal stress also impacts oxidative balance by elevating reactive oxygen species (ROS) production and altering antioxidant responses [28]. Oxidant stress induced by elevated temperatures can rapidly active antioxidant defenses in fish [29]. For instance, in *Onychostoma macrolepis* (Bleeker, 1871), the mRNA expression of SOD and CAT increased significantly within 48 h of exposure to 30 °C, indicating a rapid early-phase antioxidant response [30]. Similarly, in juvenile hybrid sturgeon, high temperatures led to MDA accumulation and elevated hepatic antioxidant enzyme activity [31]. In contrast, the current study observed a decline in SOD, CAT, and T-AOC activity, along with reduced MDA concentrations in juvenile *S. grahami* under all thermal stress conditions. This pattern suggests that prolonged exposure to elevated temperatures may overwhelm the antioxidant defense system, exhausting its capacity to neutralize sustained ROS production [32]. Interestingly, hepatic HSP70 and ATF4 protein expression was significantly upregulated only at 24 °C, possibly reflecting a specific adaptive response to moderate thermal stress at 18 °C and 21 °C [33]. Additionally, IL-1β and IL-10 levels were significantly increased only in the 18 °C group, followed by a return to control levels. This transient immune response indicates that the immune system was not significantly affected at this temperature. Similarly, the apoptosis and autophagy processes, as indicated by fluctuations in p38 and LC3, were not triggered under thermal stress at 24 °C. However, these mechanisms may become active at higher temperatures, such as 27 °C, leading to the complete mortality observed in this study.

### 4.3. Chronic Exposure to Thermal Stress Leads to Intestinal Villus Hypertrophy and Alters the Gut Microbiota Community Structure in Juvenile S. grahami

Beyond systemic physiological changes, temperature also influences intestinal morphology and microbiota composition in juvenile *S. grahami*. Optimal temperatures enhance fish feeding activity, metabolism, and intestinal villus, thereby improving nutrient absorption [34]. In this study, intestinal villus length and muscularis thickness were significantly increased in a temperature-dependent manner at 18 °C and 21 °C, potentially reflecting adaptive hypertrophy to meet increased metabolic demand. Similar adaptive changes have been reported in the juvenile butter catfish (*Ompok bimaculatus* (Bloch, 1794)) displaying increased villus size under moderate thermal stress [35]. Exceeding the critical thermal maximum leads to the epithelial cell swelling and a decrease in intestinal villi length in the cold-water fish of *Oxygymnocypris stewarti* (Lloyd, 1908) [36]. In the present study, a similar significant reduction was observed in juvenile *S. grahami* as the temperature increased from 21 °C to 24 °C, which approaches the upper thermal tolerance range of the species. Although intestinal enzyme activity was not quantified in this study, the observed morphological changes align with the literature suggesting that moderate heat may enhance intestinal enzyme activity, whereas more extreme temperature can disrupt gut integrity and inhibit key digestive enzymes such as lipase and protease [37,38].

Temperature plays a critical role in shaping the gut microbiota of poikilotherms like fish [39]. The gut microbiota is essential for maintaining host health, influencing nutrient assimilation, immune homeostasis, and physiological resilience [40]. Alpha diversity changes in juvenile *S. grahami* in this study suggests that moderate warming may enhance microbial richness, whereas excessive heat reduces diversity. This pattern aligns with previous findings in rainbow trout (*Oncorhynchus mykiss* (Walbaum, 1792)) exposed to acute heat stress [41]. Fish gut microbiota is usually dominated by the phyla of Proteobacteria, Firmicutes, and Bacteroidetes, as well as the phyla of Fusobacteriota and Actinobacteria [42]. In this study, juvenile *S. grahami* exhibited a notable increase in Fusobacteriota abundance at higher temperatures, consistent with findings in eurythermal species like *Leiocassis longirostris* (Günther, 1864) [43]. Similar temperature-driven shifts have been reported in Chinook salmon (*Oncorhynchus tshawytscha* (Walbaum, 1792)), where gut communities shifted from Proteobacteria to Fusobacteriota during 41–49 days of heat exposure [44]. *Cetobacterium* has been found to be a predominant gut microbiota genus in many freshwater and marine fish [45,46]. In this study, the increase in the relative abundance of *Cetobacterium* in juvenile *S. grahami* may have contributed to the higher proportion of the phylum Fusobacteriota. The significant increase in *Cetobacterium* spp. has been detected in the gut of reovirus-infected grass carp (*Ctenopharyngodon idellus* (Valenciennes, 1844)) [47] and *Vibrio cholerae*-infected zebrafish [48]. Identifying a single bacterial taxonomic biomarker for health status is challenging due to the wide range of factors influencing gut microbiota composition and the diversity of host-specific responses [49]. Nonetheless, the significant increase in the relative abundance of *Cetobacterium* suggests an impaired immune status in juvenile *S. grahami* under thermal stress. Furthermore, a previous study in zebrafish demonstrated that *Cetobacterium* can regulate glucose homeostasis by producing acetate, which promotes glucose-stimulated insulin secretion [50]. This mechanism may also play a role in the glucose regulation of juvenile *S. grahami* in the present study.

## 5. Conclusions

Our findings provide the first comprehensive assessment of the impacts of chronic thermal stress on juvenile *S. grahami*, covering survival, energy metabolism, oxidative balance, gut morphology, and microbial ecology. The results reveal that moderate thermal stress can trigger adaptive physiological responses, particularly in energy metabolism and antioxidant defense in juvenile *S. grahami*. Additionally, immune response, apoptosis, and autophagy were minimally affected at moderate temperatures below 24 °C. However, exposure to higher temperatures overwhelmed physiological defenses, compromising host health. Rapid energy depletion and disruption of the antioxidant system reduce the stress response capacity, while changes in the gut microbiota contribute to weakened immune function. Moreover, temperatures exceeding 30 °C may result in acute lethality in juvenile *S. grahami*, further exacerbating the species’ vulnerability in a warming climate.

In the present study, no significant growth was observed in the surviving individuals across different temperature treatment groups, which may be due to the inherently slow growth rate of juvenile *S. grahami* and the relatively short experimental duration of 30 days. Future studies should include long-term experiments across multiple developmental stages to better characterize the temperature tolerance thresholds and physiological responses of *S. grahami*. Additionally, the potential effects of decreased dissolved oxygen concentrations resulting from elevated temperatures should also be considered. Such studies will be essential for refining conservation strategies, including habitat management and adaptive measures to mitigate climate-induced thermal stress. By integrating physiological, ecological, and microbiological perspectives, these efforts can provide a robust scientific basis for safeguarding *S. grahami* in the face of ongoing climate change.

## Data Availability

Data are contained within the article or Supplementary Material.

## Supplementary Materials

The following supporting information can be downloaded at: https://www.mdpi.com/article/10.3390/ani15182749/s1, Figure S1: Original bands for each protein in western blot assay; Table S1. Commercial kits used for serum biochemical indicator measurement; Table S2. Count number of each parameter in the intestinal histological analysis; Table S3. The antibody used in the present study; Table S4. Relative intensity ratio of each band normalized to GAPDH in western blot assay; Table S5. RT-qPCR-selected genes and gene-specific primers. References [18,51,52,53,54,55] are cited in Supplementary Materials.
