# Supplementary material for: Effects of Chronic Thermal Stress on the Physiology, Metabolism, Histology, and Gut Microbiota of Juvenile Schizothorax grahami
Source: Animals (Basel). 2025 Sep 20;15(18):2749. doi: 10.3390/ani15182749 (PMC12466422; doi:10.3390/ani15182749)
Supplement: Supplementary file 1 [file animals-15-02749-s001.zip › animals-3843107-supplementary.pdf]

Table S1. Commercial kits used for serum biochemical indicator measurement

| Kits | Manufacturer | Catalog Number |
|---|---|---|
| Triglyceride assay kit | Nanjing Jiancheng Bioengineering Institute, Nanjing, China | A110-1-1 |
| Total cholesterol assay kit | Nanjing Jiancheng Bioengineering Institute, Nanjing, China | A111-1-1 |
| Glucose kit (glucose oxidase method) | Nanjing Jiancheng Bioengineering Institute, Nanjing, China | A154-1-1 |
| Total antioxidant capacity assay kit (FRAP method) | Nanjing Jiancheng Bioengineering Institute, Nanjing, China | A015-3-1 |
| Superoxide Dismutase (SOD) assay kit (WST-1 method) | Nanjing Jiancheng Bioengineering Institute, Nanjing, China | A001-3-2 |
| Cell Malondialdehyde (MDA) assay kit (Colorimetric method) | Nanjing Jiancheng Bioengineering Institute, Nanjing, China | A003-4-1 |
| Catalase (CAT) assay kit (Visible light) | Nanjing Jiancheng Bioengineering Institute, Nanjing, China | A007-1-1 |

Table S2. Count number of each parameter in the intestinal histological analysis

| Parameters | Muscularis thickness | Villi length | Villi width |
|---|---|---|---|
| Ctrl | 162 | 53 | 132 |
| 18 °C | 160 | 52 | 147 |
| 21 °C | 196 | 56 | 149 |
| 24 °C | 152 | 56 | 149 |

Table S3. The antibody used in the present study

| Antibody | Dilution Rate | Manufacturer | Catalog Number |
|---|---|---|---|
| GAPDH Monoclonal antibody | 1:50000 | Proteintech, China | 60004-1-Ig |
| Anti-ATF-4 Rabbit pAb | 1:1000 | Servicebio, China | GB111157 |
| Recombinant Anti-HSP70 antibody（Mouse mAb） | 1:1000 | Servicebio, China | GB15241 |
| IL-1 Beta Polyclonal antibody | 1:5000 | Proteintech, China | 16806-1-AP |
| IL-10 Mouse McAb | 1:5000 | Proteintech, China | 60269-1-Ig |
| Anti-LC3A/B Rabbit pAb | 1:1000 | Servicebio, China | GB11124 |
| Recombinant Anti-p38 antibody（Rabbit mAb） | 1:1000 | Servicebio, China | GB154685 |
| HRP-conjugated Goat Anti-Mouse IgG(H+L) | 1:10000 | Proteintech, China | SA00001-1 |
| HRP-conjugated Goat Anti-Rabbit IgG(H+L) | 1:10000 | Proteintech, China | SA00001-2 |

Table S4. Relative intensity ratio of each band normalized to GAPDH in western blot assay

| | HSP70 | p38 | ATF4 | IL-1β | IL-10 | LC3 |
|---|---|---|---|---|---|---|
| Ctrl | 0.99±0.01[b] | 1.00±0.01[b] | 0.99±0.01[b] | 0.99±0.01[b] | 1.00±0.01[b] | 1.00±0.01[a] |
| 18°C | 1.72±0.51[b] | 1.50±0.23[a] | 1.43±0.08[b] | 14.91±6.19[a] | 2.31±0.52[a] | 0.77±0.08[b] |
| 21°C | 1.87±0.79[b] | 0.47±0.06[c] | 0.85±0.24[b] | 0.57±0.11[b] | 1.18±0.22[b] | 0.93±0.06[a] |
| 24°C | 17.19±8.39[a] | 0.92±0.11[b] | 4.58±1.70[a] | 2.75±0.99[b] | 0.78±0.10[b] | 0.68±0.02[b] |

Note: Intensity ratio with different letters indicated significant differences ($p < 0.05$).

Table S5. RT-qPCR-selected genes and gene-specific primers

| Gene name | Primer sequences (5'- 3') | Ref. |
| --- | --- | --- |
| *lpl* | F: GCAACAACACTACCCTACATC<br>R: GGTGAGAAGACCAGCAAT | [51] |
| *β-actin* | F: GATGGACTCTGGTGATGGTGTGAC<br>R: TTCTCTTTCGGCTGTGGTGGTG | [18] |
| *hsp70* | F: TGAGAACATCAACGAGCCCA<br>R: TTGTCAAAGTCCTCCCCACC | [18] |
| *igf1* | F: TGCAGTTTGTGTGTGGAGAC<br>R: CACAGCTCTGAAAGCAGCAT | [18] |
| *g6pc1a* | F: CCTGAAGGCTCTCGGTGTGGAT<br>R: ACTTGCGAACGGTGTGGTGTC | [52] |
| *cebpa* | F: GCGTCTAAGATGAGGGAGTC<br>R: TGAGTATCCAAGGCACAAGG | [53] |
| *pparaa* | F: CTCCGCCTTTCGTCATCC<br>R: CAGTAGAGTCACCTGGTCGTTG | [51] |
| *pik3* | F: GAAGATGACGACTGGAGAG<br>R: GCCTGTAGTGACTGATGAG | [54] |
| *gk* | F: AGAGGAAGTGATGAGGAGAA<br>R: GTGGAGCGGACATAAGTG | [52] |
| *lc3b* | F: AGCAGCGGGTGGAGGATGTA<br>R: CCTCAGAAATGGCGGTGGAC | [55] |

Figure S1. Original bands for each protein in western blot assay

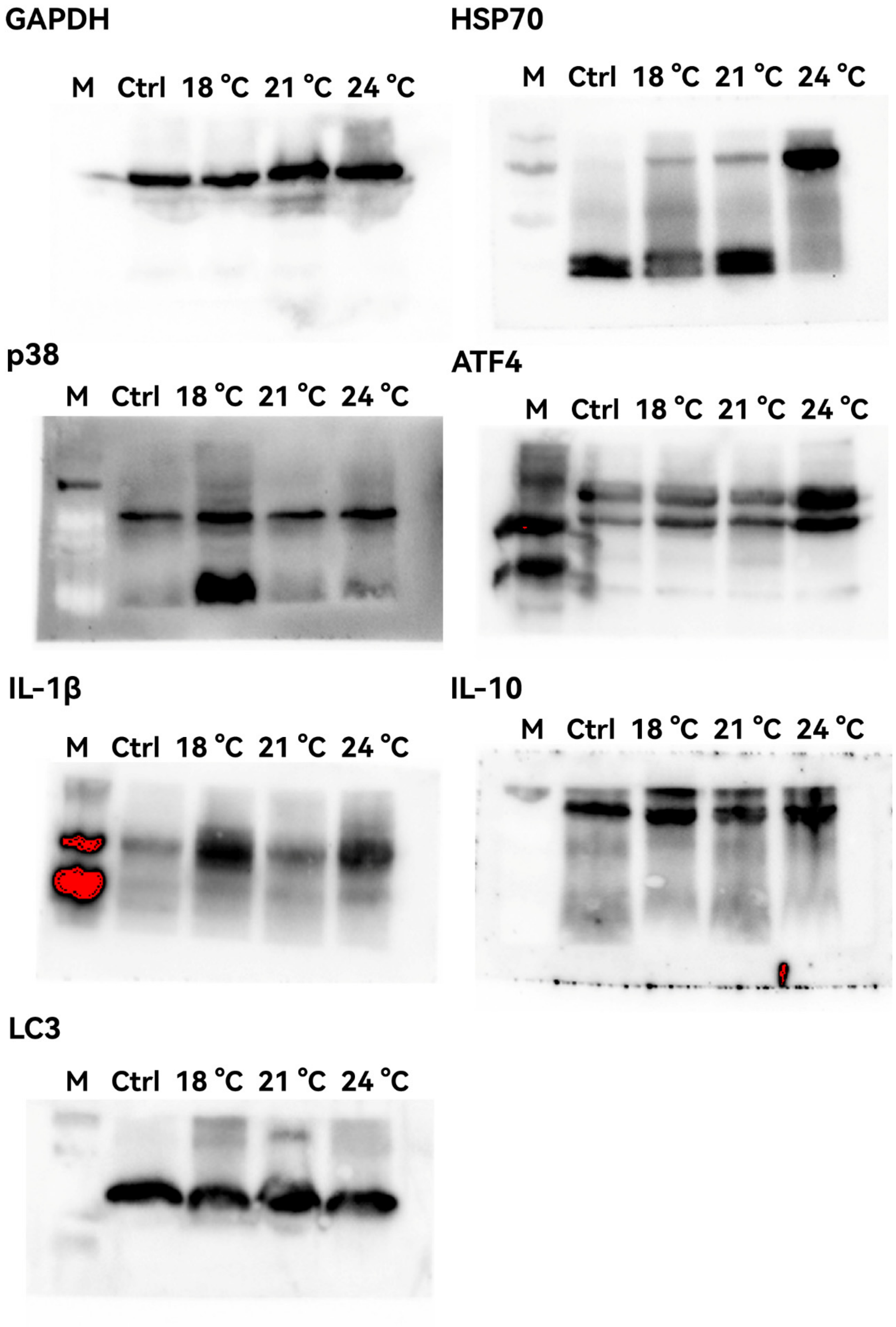


M: Prestained Protein Marker (8-200 kDa)

Reference


51. Zhao, L.L., Sun, J.L., Liang, J., Liu, Q., Luo, J., Li, Z.Q., Yan, T.M., Zhou, J., and Yang, S. Enhancing lipid metabolism and inducing antioxidant and immune responses to adapt to acute hypoxic stress in *Schizothorax prenanti*. *Aquaculture* **2020**, *519*, 734933. https://doi.org/https://doi.org/10.1016/j.aquaculture.2020.734933

18. Li, S., Guo, H., Chen, Z., Jiang, Y., Shen, J., Pang, X., and Li, Y. Effects of acclimation temperature regime on the thermal tolerance, growth performance and gene expression of a cold-water fish, *Schizothorax prenanti*. *J. Therm. Biol.* **2021**, *98*, 102918. https://doi.org/https://doi.org/10.1016/j.jtherbio.2021.102918

52. Zhang, R., Mo, Q., Wang, A., Zhang, X., Chen, C., Wang, L., and Wang, Y. Axin interactor, dorsalization-associated (AIDA) protein promotes appetite and regulates hepatic glycolipid metabolism in *Schizothorax prenanti*. *Aquaculture* **2025**, *595*, 741549. https://doi.org/https://doi.org/10.1016/j.aquaculture.2024.741549

53. Silvestri, C., Martella, A., Poloso, N.J., Piscitelli, F., Capasso, R., Izzo, A., Woodward, D.F., and Di Marzo, V. Anandamide-derived prostamide F2α negatively regulates adipogenesis. *J. Biol. Chem.* **2013**, *288* (32), 23307-23321. https://doi.org/https://doi.org/10.1074/jbc.M113.489906

54. Zhang, J.Q., Zheng, S.F., Wang, S.C., Liu, Q.Q., and Xu, S.W. Cadmium-induced oxidative stress promotes apoptosis and necrosis through the regulation of the miR-216a-PI3K/AKT axis in common carp lymphocytes and antagonized by selenium. *Chemosphere* **2020**, *258*. https://doi.org/10.1016/j.chemosphere.2020.127341

55. Wang, S., Zheng, S., Zhang, Q., Yang, Z., Yin, K., and Xu, S. Atrazine hinders PMA-induced neutrophil extracellular traps in carp via the promotion of apoptosis and inhibition of ROS burst, autophagy and glycolysis. *Environ. Pollut.* **2018**, *243*, 282-291. https://doi.org/https://doi.org/10.1016/j.envpol.2018.08.070
